# Partnership for productive development of biosimilar products: perspectives of access to biological products in the Brazilian market

**DOI:** 10.1590/S1679-45082018RW4175

**Published:** 2018-09-10

**Authors:** Morton Aaron Scheinberg, Paulo Antonio Oldani Felix, Igor Age Kos, Maurício De Angelo Andrade, Valderilio Feijó Azevedo

**Affiliations:** 1 Hospital Israelita Albert Einstein, São Paulo, SP, Brazil; 2 Hospital Federal dos Servidores do Estado, Rio de Janeiro, RJ, Brazil; 3 Universidade Federal do Paraná, Curitiba, PR, Brazil; 4 Laboratórios Pfizer Ltda, São Paulo, SP, Brazil

**Keywords:** Biosimilar pharmaceuticals, Public-private sector partnerships, Health services accessibility, Medicamentos biossimilares, Parcerias público-privadas, Acesso aos serviços de saúde

## Abstract

The manufacturing process for biological products is complex, expensive and critical to the final product, with an impact on their efficacy and safety. They have been increasingly used to treat several diseases, and account for approximately 50% of the yearly budget for the Brazilian public health system. As the patents of biological products expire, several biosimilars are developed. However, there are concerns regarding their efficacy and safety; therefore, the regulatory agencies establish rules to approve and monitor these products. In Brazil, partnership programs between national government-owned companies and private technology holders have been implemented, aiming at knowledge sharing, capacity-building and technological transfer. Such partnerships locally promote manufacturing of these strategic drugs at reduced costs to the public health system. These agreements offer mutual advantages to both the government and patent holders: for the former, a biotechnological development flow is established and enables potential cost reduction and self-sufficient production; whereas for the latter, exclusive sales of the product are ensured during technological transfer, for a fixed period.

## RESUMO

O processo de manufatura de produtos biológicos é complexo, oneroso e crítico para o produto final, com impacto em sua eficácia e segurança. Seu uso está sendo cada vez mais ampliado no tratamento de diversas doenças, e cerca de 50% do orçamento anual do sistema de saúde público brasileiro é consumido por tais produtos. Com o término da proteção de patentes de produtos biológicos diversos, estão sendo desenvolvidos os biossimilares. Porém, há preocupações relacionadas com sua eficácia e segurança, fazendo com que os órgãos reguladores criem regulamentações para sua aprovação e monitoramento. No Brasil, estão sendo implantados programas de parceria entre laboratórios públicos nacionais e laboratórios detentores de tecnologia, objetivando a obtenção de conhecimento, capacitação profissional e transferência desta tecnologia. Tais parcerias visam à produção local destes medicamentos estratégicos a um custo reduzido para o Sistema Único de Saúde. Os acordos oferecem vantagens mútuas para o governo e o laboratório detentor da patente do produto biológico: ao primeiro, estabelece-se um fluxo de desenvolvimento biotecnológico, que possibilita potencial redução de custos e autossuficiência na produção, enquanto ao segundo garante-se a exclusividade da venda do produto durante a transferência da tecnologia por um prazo estabelecido.

## INTRODUCTION

The advent of biopharmaceuticals has completely transformed the treatment of different conditions. Their manufacturing process is complex and costly, requiring living organisms that produce large and complex molecular structures, and small changes in the conception and execution of this process may directly affect their efficacy and safety.^(^ ^1^ ^)^ The process is so critical to the final product that most manufacturers file for patents related to the manufacturing process and not necessarily the biologic agent *per se.*

As the patents of different biologics expire, companies are allowed to develop and sell these products. Due to particularities in the production process, which is not publicly shared and disclosed by the originator company, the creation of an identical copy is virtually impossible, and this prevents the experience with generics (obtained by chemical synthesis) to be applicable to biologics. This gives rise to relevant questions and concerns related with efficacy, safety and immunogenicity of these products.^(^ ^1^ ^-^ ^3^ ^)^

Since biologics use up a considerable portion of the national health care budgets, there is high financial pressure for the adoption of very similar copies, known as biosimilars.^(^ ^3^ ^)^ Considering these facts, the regulatory bodies and medical associations worldwide are challenged with establishing rules to determine the similarity degree of a biosimilar with its reference product, to ensure they have the same quality, efficacy and safety profile, which would allow for their approval and marketing.

To discuss the most relevant topics related with the particularities of introducing biosimilars in the Brazilian market, we conducted a qualitative search of major health search databases (PubMed and LILACS), as well as databases of national and international health authorities and organizations (Brazilian National Health Surveillance Agency - ANVISA, Brazilian Ministry of Health, World Health Organization - WHO, Food and Drug Administration - FDA, and European Medicines Agency - EMA).

### Global Recommendations for Evaluating Biosimilars

In 2009, the WHO published the Guidelines on Evaluation of Similar Biotherapeutic Products (SBP),^(^ ^4^ ^)^ which is used by different regulatory bodies as a basis for drafting regulations. To assess similarity, the first step is the complete physical-chemical and biological characterizations of the biosimilar, in a head-to-head comparison with the reference product.

Besides this physical-chemical characterization, the binding affinity to cell receptors is evaluated based on animal trials and studies, including pharmacodynamics and toxicity. The methods used to determine the comparability between biosimilars and their reference product must be sufficiently selective and specific to detect any differences between both. The relevance of these differences can only be verified in pre-clinical and clinical trials. Therefore, a risk-based approach has been recommended for assessing biosimilarity,^(^ ^5^ ^)^ since risk is reduced by pre-setting, with relative approximation, the comparison statistics between the innovator product and the biosimilar. Furthermore, differences against the innovator product must be detected by independent phase III outcome studies, preceded by mandatory phase I pharmacokinetic and pharmacodynamic studies.^(^ ^5^ ^)^ Finally, the route of administration and dosage of a biosimilar must also be the same as those of the reference product.

The efficacy and safety of biosimilars must resemble those of the reference product, as demonstrated by randomized, double-blind, controlled (with the reference product) clinical trials. The preferred design for data comparison in said studies is the equivalence trial (with definition of upper and lower comparative limits). In other circumstances, non-inferiority trials can be used.^(^ ^5^ ^)^

The equivalence/non-inferiority margin must be previously specified and justified to regulatory authorities, based on the clinical relevance, and the differences detected in treatment effects must be acceptable to the medical community, without any detrimental impact on patient care.

Oftentimes, biologics are immunogenic. This entails an evaluation of the potential immunogenicity of a biosimilar and, consequently, of its safety and efficacy, with correct determination of the prevalence of antidrug antibodies. This evaluation must also be carried out for the reference product.^(^ ^6^ ^)^

After the biosimilarity has been verified, there are still some controversial issues, such as the extrapolation of indications, the nomenclature of the new drug, and interchangeability, all based on the fact that the products are not identical molecules.

The extrapolation for other indications is a procedure allowed by the FDA and EMA, provided that the mechanism of action is the same for the indications considered, and the extrapolated indication does not include the pediatric population. However, this leads to countries taking different stands, since most of the conditions treated with biologics do not have their pathogenesis totally understood, which makes it impossible to demonstrate that the mechanism of action is the same for different indications.^(^ ^7^ ^,^ ^8^ ^)^

Post-marketing pharmacovigilance is also essential to identify and monitor rare or uncommon adverse events, in addition to other issues related with efficacy. However, the pharmacovigilance process can be greatly affected by the lack of definition of a biosimilar nomenclature. The use of the name of the main ingredient (as we see with generics) does not apply to biosimilars, which are very similar molecules, but not identical. Currently, to work around this issue, both the WHO and FDA recommend that a biologic qualifier (BQ) be added to the generic name of the reference product, allowing for the distinction between the reference and the biosimilar at issue.^(^ ^6^ ^,^ ^9^ ^,^ ^10^ ^)^

Confirmation of biosimilarity does not mean, however, that the products are interchangeable, and regulatory agencies do not yet have a definitive solution to this matter. This led the EMA to leave this decision to each of its member-countries, independently, since this can affect medical prescriptions. On the other hand, the FDA has been requiring additional data for interchangeability approval.

Furthermore, there is the concept of “automatic substitution”, which takes place at the time of drug dispensation. If approved, it allows one drug to be substituted for another, without the prescriber or the patient knowing, which makes it quite difficult to identify the product causing potential adverse events over the course of a treatment.^(^ ^11^ ^)^

### Legislative situation in Brazil

It was only in 2010 that ANVISA published its Collegiate Board Resolution (RDC) 55/2010 on this matter, establishing the criteria for biosimilar approval in the country. Before that, products were approved without any specific regulations and, as of 2002, authorities started to require the conduction of clinical trials for registration renewal of biologic products already in the market.^(^ ^12^ ^)^ Collegiate Board Resolution 55/2010 provides two routes for biosimilarity approval: one known as “comparability”, and another called “individual development”.^(^ ^13^ ^)^

In the same resolution, the nomenclature used, differently from most scientific studies, calls biosimilars “biologic products” and reference products “new biologics”, which can be confusing.^(^ ^13^ ^)^ The comparative route is nearly identical to that described in the WHO document, which calls them Similar Biotherapeutic Products (SBP) and is, therefore, more rigorous and requires phase I and phase III comparison studies with the Reference Biotherapeutic Product (RBP), in addition to allowing extrapolation for other indications. The “individual development” route precludes the comparability exercise with a shorter dossier, which creates some concern regarding its use, particularly in the regulation of biosimilar monoclonal antibodies. However, the extrapolation of indications, which is an important and controversial point about biosimilar products, is not allowed in this route.

Thus, copies licensed using the comparability route can truly be called “biosimilars”.^(^ ^13^ ^)^ Table 1 shows an overall comparison of the two routes of RDC 55/2010 and those recommended in the WHO document.

**Table 1:** Comparison of recommendations from the World Health Organization and Brazilian requirements, in the two routes of biosimilar approval

	World Health Organization	Brazil - individual development	Brazil - comparability
Chemistry, manufacturing and documentation control	Comparative data only	As per development standards	Comparative data only
Pre-clinical trials	Comparative data with the reference product only	Comparative data, with a few exceptions	Comparative data with the reference product
Phase I clinical trials	Comparative pharmacokinetic data	There is no need for comparative data	Comparative pharmacokinetic data
Phase III clinical trials	Comparative efficacy and safety data, tested for a condition considered as a sensitivity model for comparison purposes	Comparative data, with a few exceptions	Comparative efficacy and safety data, similar to the recommendations of the World Health Organization
Extrapolation of indications	Yes	No	Yes
Interchangeability	Suggests data evaluation for interchangeability	No	Nothing on this matter
Biosimilar nomenclature	Suggests a Common International Nomenclature followed by 4 random letters	Not defined	Not defined
Pharmacovigilance system	Robust, similar to that of the reference product	As per development standards	Robust, similar to that of the reference product

### Partnership for Productive Development

Partnership for Productive Development (PPD) is a program between national public pharmaceutical companies and private technology holders, either national or international, aiming at knowledge sharing, capacitybuilding and technology transfer for local production of strategic drugs, reducing costs to the public health care system (SUS - *Sistema Único de Saúde*).^(^ ^14^ ^)^

Recently, in 2013, the program was expanded with the approval of several PPDs for high-cost biologics, allowing for lower prices in comparison with the private market. The rules of the PPD program were revised on November 12th, 2014, with the publication of a new ordinance (Ordinance 2,531/2014) redefining the criteria and rules for establishing and monitoring PPDs.^(^ ^15^ ^)^

The estimated financial benefits of this program are enormous, since 50% of the annual drug budget of the Ministry of Health is used up by this class of drugs (*e.g.* adalimumab, etanercept and infliximab, in decreasing order), serving a much smaller number of patients when compared with diabetes or hypertension medications.^(^ ^16^ ^)^

The PPD proposals are jointly developed by the patent holder and the public pharmaceutical company, and presented by the latter to the competent department of the Ministry of Health (Science, Technology and Strategic Inputs Department), at specific times during the year. The projects include information on the participants, the product, its development history, productive process and technology employed, as well as the investments required.^(^ ^15^ ^)^

The decision-making process is as per the rite described in Ordinance 2,531, including a technical analysis by a Technical Evaluation Committee, whose report is submitted for approval by the Board of Directors, with results announced at the meetings of the Health Industry Executive Group (GECIS).^(^ ^15^ ^)^ In accordance with Ordinance 2,531, the duration of the PPD must not exceed 10 years. An important point is that the ordinance also determines that the master cell bank of the original product be mandatorily transferred to the public organization participating in the PPD, for storage and future use in product manufacturing.^(^ ^15^ ^)^

For the public organization in Brazil to have access to all the technology and support required to manufacture the biologic, the Ministry of Health will buy the product ready-made, exclusively from the manufacturer, as part of the development of the PPD project, including registration with ANVISA. The first acquisition takes place after the technology transfer contract is executed between the technology holder and the public pharmaceutical company, and subsequent purchases may take place only after the start of the technology transfer to the public organization is confirmed.

Thus, PPDs seems to offer a win-win opportunity for the government and private companies. For the first, a biotechnological development flow is established, allowing for potential cost reduction and self-sufficient production, whereas for the second, exclusive sales rights are ensured during the period of technology transfer.

The first PPD and technology transfer of a monoclonal antibody involves the private company Janssen-Cilag Farmacêutica Ltda., the technology holder, Bionovis S.A., a national biotechnology company, and the public pharmaceutical company Bio-Manguinhos. The biological product is Remicade^®^ (infliximab), which has been available in the Brazilian market since 1998.^(^ ^17^ ^)^ According to data from Bio-Manguinhos, more than 80% of infliximab vials purchased by the Ministry of Health in 2015 came from the program, corresponding to a total of approximately 180 thousand vials and BRL175 million.^(^ ^17^ ^)^

Bionovis S.A. will be responsible for production in the private sector. This company is a joint venture between companies Aché, EMS, Hypera Pharma and União Química Farmaceutica Nacional S.A, with a manufacturing site under construction in the city of Valinhos (SP). We must highlight that the market of choice of the treatment will undergo great transformations with the advent of biosimilars and oral small molecules (targeted therapies), since said drugs have already been included or are under consideration for inclusion in the so-called Clinical Protocols and Therapeutic Guidelines (PCDT - *Protocolos Clínicos e Diretrizes Terapêuticas*).^(^ ^17^ ^)^

Other biologics, also subject to PPDs, are biosimilars of widely used innovator biologics, well established in the Brazilian market. These include rituximab and adalimumab, for which PPDs have been established between other public companies and technology holders and their national private partners.^(^ ^16^ ^)^ This is the case of national company Orygen Biotecnologia, another joint venture between Eurofarma and Biolab, including Pfizer as a technology transfer partner, as well as national public companies Bahiafarma (infliximab and rituximab) and Bio-Manguinhos (adalimumab). In addition to the abovementioned PPDs, there is another one for bevacizumab, an important anticancer drug, also in partnership with Bio-Manguinhos.^(^ ^17^ ^)^

The first biosimilar sold in Brazil was that of the monoclonal antibody infliximab, called Remsima, available since 2016, however with no access to the public market through a PPD. Despite having been the first biologic with a good efficacy and safety profile in rheumatology, dermatology and gastroenterology indications, its prescription by specialists has been considerably decreasing both in Brazil and abroad, possibly due to its intravenous administration.

The etanercept biosimilar developed in compliance with the rigorous requirements of renowned regulators, such as FDA and EMA, is now approved in Europe under the trade name Benepali, and as Brenzys in Canada, Australia and Korea.^(^ ^18^ ^,^ ^19^ ^)^ Differently from the intended copies present in other Latin American markets (Etanar and Infinitam), this biosimilar is supposed to be introduced in Brazil by the company Samsung-Bioepis, a joint venture of Korean, European and North-American origin, and distributed by Merck Sharp & Dohme. However, due to global trade agreements between the original manufacturers and their partners, the etanercept biosimilar is not included in the PPD program, and can be supplied to the public market through call for tenders and buying processes, just like any other product not included in the PPD program.

## COMMENTS

The inherent characteristics of biological products do not allow the experience gained with generic drugs to be used in the making of “copies” known as biosimilars. Several questions and concerns have emerged over time, some still open and not clearly solved. Whereas the marketing of biosimilars is expected to reduce the usually high costs of treatments with biologics, it should be done in a way as to avoid any detrimental impact to patients, when it comes to safety and efficacy. A post-marketing pharmacovigilance process allowing for distinction between reference products and biosimilars and monitoring of their effects is essential for this goal to be achieved.

Regulatory agencies are adapting as more data become available, and Brazil has also been updating its regulations, which are currently not so different from their international versions. In addition to regulations for approval and monitoring, the Ministry of Health has developed a plan to improve access to products, advocating that national production of biologics can generate savings to the country and allow for lower prices. This could potentially have a positive effect on the Brazilian trade balance, generating taxes and revenue to the country.

The impact on the production chain will be even more relevant, involving suppliers of inputs, raw materials, and equipment, generating jobs, training the work force to ensure drug quality, and fostering infrastructure investments.

The recent introduction of biosimilars has created a new context, requiring the development of new regulations and trade processes. Several questions remain open, and regulators must carefully monitor and adapt their procedures in response to new data. The situation in Brazil is currently harmonized with the rest of the world.
